# Chemical Constituents, Antioxidant, and Enzyme Inhibitory Activities Supported by In-Silico Study of *n*-Hexane Extract and Essential Oil of Guava Leaves

**DOI:** 10.3390/molecules27248979

**Published:** 2022-12-16

**Authors:** Shaza H. Aly, Omayma A. Eldahshan, Sara T. Al-Rashood, Faizah A. Binjubair, Mahmoud A. El Hassab, Wagdy M. Eldehna, Stefano Dall’Acqua, Gokhan Zengin

**Affiliations:** 1 Department of Pharmacognosy, Faculty of Pharmacy, Badr University in Cairo (BUC), Cairo 11829, Egypt; 2 Pharmacognosy Department, Faculty of Pharmacy, Ain Shams University, Cairo 11566, Egypt; 3 Center for Drug Discovery Research and Development, Ain Shams University, Cairo 11566, Egypt; 4 Department of Pharmaceutical Chemistry, College of Pharmacy, King Saud University, P.O. Box 2457, Riyadh 11451, Saudi Arabia; 5 Department of Medicinal Chemistry, Faculty of Pharmacy, King Salman International University (KSIU), South Sinai 46612, Egypt; 6 Department of Pharmaceutical Chemistry, Faculty of Pharmacy, Kafrelsheikh University, Kafrelsheikh 33516, Egypt; 7 School of Biotechnology, Badr University in Cairo, Badr City, Cairo 11829, Egypt; 8 Department of Pharmaceutical and Pharmacological Sciences, University of Padova, 35131 Padova, Italy; 9 Department of Biology, Science Faculty, Selcuk University, Konya 42130, Turkey

**Keywords:** antioxidants, cholinesterase, enzyme inhibition, GC/MS, Myrtaceae, *Psidium guajava*, tyrosinase

## Abstract

*Psidium guajava* (Guava tree) is one of the most widely known species in the family Myrtaceae. The Guava tree has been reported for its potential antioxidant, anti-inflammatory, antimicrobial, and cytotoxic activities. In the current study, the chemical compositions of the *n*-hexane extract and the essential oil of *P. guajava* were investigated using the GC/MS analysis, along with an evaluation of their antioxidant potential, and an investigation into the enzyme inhibition of acetylcholinesterase (AChE), butyrylcholinesterase (BchE), tyrosinase, α-amylase, and α-glucosidase. Moreover, molecular docking of the major identified active sites of the target enzymes were investigated. The chemical characterization of the *n*-hexane extract and essential oil revealed that squalene (9.76%), *α*-tocopherol (8.53%), and *γ*-sitosterol (3.90%) are the major compounds in the *n*-hexane extract. In contrast, the major constituents of the essential oil are D-limonene (36.68%) and viridiflorol (9.68%). The *n*-hexane extract showed more antioxidant potential in the cupric reducing antioxidant capacity (CUPRAC), the ferric reducing power (FRAP), and the metal chelating ability (MCA) assays, equivalent to 70.80 ± 1.46 mg TE/g, 26.01 ± 0.97 mg TE/g, and 24.83 ± 0.35 mg EDTAE/g, respectively. In the phosphomolybdenum (PM) assay, the essential oil showed more antioxidant activity equivalent to 2.58 ± 0.14 mmol TE/g. The essential oil demonstrated a potent BChE and tyrosinase inhibitory ability at 6.85 ± 0.03 mg GALAE/g and 61.70 ± 3.21 mg KAE/g, respectively. The *α*-amylase, and *α*-glucosidase inhibitory activity of the *n*-hexane extract and the essential oil varied from 0.52 to 1.49 mmol ACAE/g. Additionally, the molecular docking study revealed that the major compounds achieved acceptable binding scores upon docking with the tested enzymes. Consequently, the *P. guajava n*-hexane extract and oil can be used as a promising candidate for the development of novel treatment strategies for oxidative stress, neurodegeneration, and diabetes mellitus diseases.

## 1. Introduction

Secondary metabolites from natural sources are regarded as a provenance for alleviating and curing a plethora of ailments [1,2,3,4,5]. Neurodegenerative diseases refer to different conditions in the breakdown and damage to the central nervous system (CNS), such as dementia and Alzheimer’s disease (AD) with an impact on more than 20 million people globally. Among the therapeutic approaches followed for the management of AD are the development of therapeutic techniques based on the inhibition of key enzymes involved in the pathogenesis of the disease as antioxidant and anticholinesterase agents (AChE) [6,7,8]. Where, the use of enzyme inhibitors has significant implications for disease prevention and therapy. In addition, the management of diabetes is efficiently based on the inhibition of two enzymes *α*-amylase and *α*-glucosidase. The efficient strategy to control blood sugar levels is to delay the breakdown of carbohydrates in the small intestine in order to diminish the postprandial increase in blood glucose [9,10].The use of synthetic drugs have many diverse side effects, such as hypoglycemia, edema, mild anemia, hepatotoxicity, and weight gain [11,12]; therefore, there is a great demand to explore a new agent from natural sources for the management of neurodegenerative diseases, oxidative stress, and diabetes.

The family Myrtaceae is one of the most important commercial families in the world, it has great economical and nutritional values that are linked to the management of different illnesses [13]. It is a diverse botanical family that comprises characteristic genera, including, but not limited to, *Syzygium*, *Eucalyptus*, *Myricaria*, *Melaleuca*, *Eugenia*, *Myrtus,* and *Pisidium* [14,15,16,17]. It has an economic potential value due to its pleasant sensory properties and bioactive constituents, and it is considered as a continuous source of antioxidant agents [18].

One of the most known genera of the Myrtaceae family is the genus *Pisidium,* which includes about 150 species [19]. *Pisidium guajava* L. is the most famous species, having the common name Guava tree, it is an evergreen shrub with curved wide spreading branches and bears opposite green leaves with small petioles [20,21,22]. It is distributed throughout the world’s tropical and subtropical regions [23,24]. It has a long history in traditional medicine all over the world as treatment for diarrhea, diabetes, cough, stomach pain, dysentery, toothache, indigestion, constipation, fever, and wound healing; it is notable that different parts, such as leaves, flowers and barks are involved in traditional uses in the form of decoction and infusions [25,26,27]. Another point is the outstanding pharmacological properties of *P. guajava,* including, but not limited to, antioxidant, anti-inflammatory, antimicrobial, cytotoxic, analgesic, cardioprotective, hepato-protective, and antidiabetic activities [28,29,30,31].

Regarding the phytochemical composition of *P. guajava*, it is a rich source of flavonoids, phenolic acids, triterpenoids, vitamins, and minerals [29,32]. Moreover, the volatile components comprise mainly sesquiterpenes and monoterpenes [33,34]. The nutritional value of *P. guajava* is particularly noticeable as a functional food ingredient [32]. The fruit is the richest part with a high vitamin C content, so it is commonly used for colds and infections. Regarding the essential oil of *P. guajava,* it has been demonstrated to provide various health benefits, such as antimicrobial, antinociceptive, anti-inflammatory, insect repellent, and insecticidal activities [35].

There have been several reports concerning the biological activities of different *Pisidium* species [36]. For example, de Souza Cardoso et al. (2018) reported the antidiabetic effects of phenolic compounds, especially anthocyanins in *P. cattleyanum* fruits [37]. Another study revealed the analgesic activity of the hydroalcoholic extract of the leaves of *P. cattleyanum* [38]. Another study reported that the methanol extract of *P. sartorianum* fruit pulp displayed a remarkable antifungal activity [39]. Therefore, we are interested in conducting a comprehensive study to compare the volatile components of the *n*-hexane extract and the essential oil of *P. guajava* based on the GC/MS analyzes. Additionally, we want to investigate their antioxidant and enzyme inhibitory activities based on five key enzymes (acetyl/butyryl-cholinesterase, tyrosinase, α-amylase, and α-glucosidase), to assess the efficacy of the oil and the *n*-hexane extract as enzyme inhibitors. In addition, molecular docking studies were performed to demonstrate the possible mechanism of action of the main compounds identified in *n*-hexane and essential oils and how they exert their biological activities.

## 2. Results and Discussion

### 2.1. GC/MS Analysis of the n-Hexane Extract and Essential Oil of Psidium guajava

**Figure 1:**
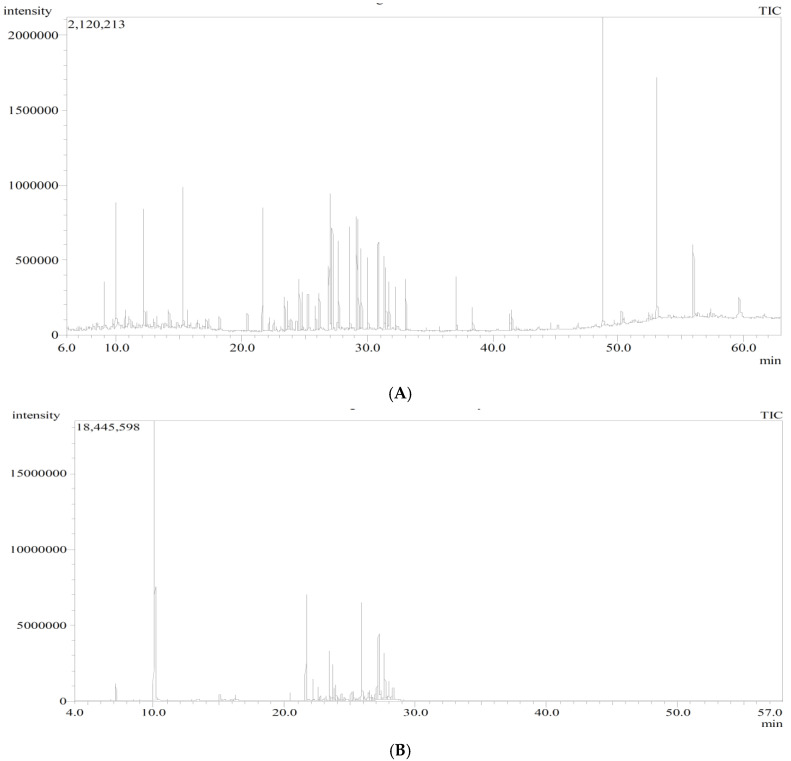
GC chromatogram of (**A**) *n*-hexane extract and (**B**) essential oil of *P. guajava* leaves.

**Figure 2:**
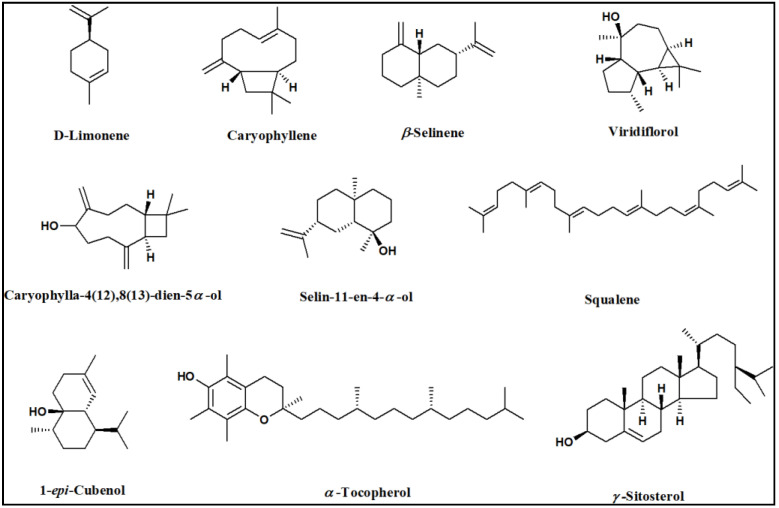
Chemical structures of the major constituents identified in the *n*-hexane extract and essential oil of *Psidium guajava* leaves using GC/MS analysis.

**Figure 3:**
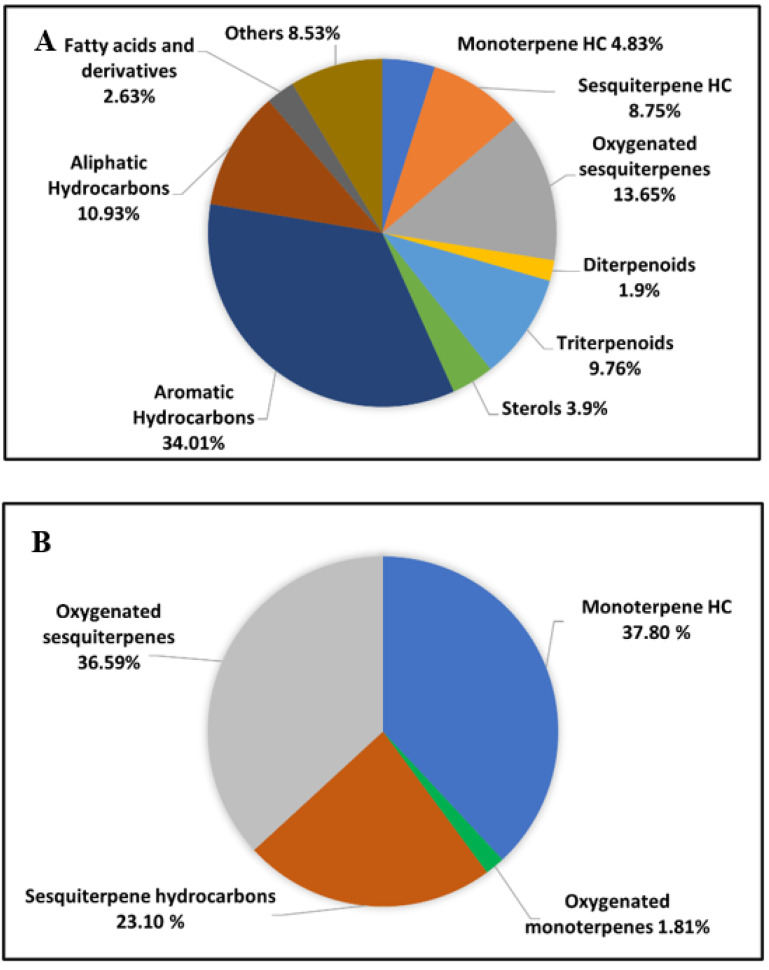
Pie charts demonstrate the distribution of volatile components as a percentage within (**A**) *n*-Hexane extract and (**B**) essential oil of *P. guajava* leaves.

The results of the GC/MS analysis of the *n*-hexane extract and the essential oil of *P. guajava* are represented in Figure 1 and Table 1. The chemical characterization of the *n*-hexane extract and the essential oil revealed the identification of 40 compounds and 39 compounds accounting for (98.89%) and (99.30%), respectively. The *n*-hexane extract was found to be rich in hydrocarbons; aromatic (34.01%) and aliphatic (10.93%); followed by oxygenated sesquiterpenes (13.65%) and sesquiterpene hydrocarbons (8.75%). On the other hand, the essential oil showed a high percentage of monoterpene hydrocarbons (37.80%) and oxygenated sesquiterpenes (36.59%), followed by sesquiterpene hydrocarbons (23.10%). In the *n*-hexane extract, squalene and *α*-tocopherol were the major compounds accounting for (9.76%) and (8.53%), respectively, followed by D-limonene (4.83%), 1-*epi*-cubenol (4.51%), *n*-dodecane (4.15%), *γ*-sitosterol (3.90%), and *β*-caryophyllene (3.80%). Regarding the essential oil, it showed a high percentage of D-limonene (36.68%), followed by viridiflorol (9.68%), *β*-caryophyllene (8.41%), caryophylla-4(12),8(13)-dien-5*α*-ol (6.48%), selin-11-en-4-*α*-ol (6.35%), and *β*-selinene (4.10%). It is worth mentioning that D-limonene and *β*-caryophyllene were common major compounds in both the *n*-hexane extract and the essential oil of *P. guajava*. The chemical structures of the major constituents and the distribution of volatile components as a percentage within the *n*-hexane extract and the essential oil of *P. guajava* leaves are illustrated in Figure 2 and Figure 3, respectively.

Many reports have been conducted on the essential oil compositions of *P. guajava* from varied geographical sources [30,33,40]. The major identified compounds in the essential oil isolated from the leaves collected from Brazil were *β*-caryophyllene, *α*-humulene, aromadendrene oxide, *δ*-selinene, and selin-11-en-4*α*-ol [24]. In contrast, the essential oil from the leaves collected from another source in India showed that *β*-caryophyllene, L-calamenene, (-)-globulol, and *α*-copaene were the major constituents [30]. Moreover, the chemical composition of the *n*-hexane extract of *P. guajava,* collected from Pakistan, showed a high content of vitamin E, squalene, caryophyllene, and *γ*-sitosterol [25]. Another study by Arian et al. reported that the essential oil of *P. guajava* leaves collected from Pakistan was a rich source of *β*-caryophyllene, globulol, and *trans*-nerolidol [33]. Regarding, the previous studies into the composition of the essential oil of *P. guajava* leaves indicated wide variations relative to the different locations of collection.

### 2.2. Total Phenolic and Flavonoid Content of the n-Hexane Extract of P. guajava Leaves

Phenolics compounds are present in most natural products that induce many biological activities [41,42,43,44]. The total phenolic and flavonoid content in the *n*-hexane extract of *P. guajava* leaves, was quantitatively determined, according to Zengin and Aktumsek, 2014 [45]. The Phenolic and flavonoid contents were measured as gallic acid, and rutin equivalents, respectively. The presence of 32.62 ± 0.19 mg GAE/g (gallic acid equivalent) per mg of *P. guajava n*-hexane extract was recorded for the total phenolics content. While the presence of 2.05 ± 0.14 mg RE/g (rutin equivalent) was recorded for the total flavonoids content. The results established the presence of considerable amounts of phenolics in the *n*-hexane extract.

### 2.3. Antioxidant Potential of the n-Hexane Extract and Essential Oil Isolated from P. guajava Leaves

Natural antioxidants, especially polyphenols, are becoming increasingly popular due to their beneficial effects on human health. Consequently, plant polyphenols may be able to mitigate the negative effects of oxidative stress, which has been linked to a variety of pathological processes, such as cancer, kidney disease, cardiovascular disease, neurodegeneration, age-related diseases, and diabetes [46,47,48,49,50,51]. Many reports revealed the potential of different essential oils as antioxidant agents [52], including but not limited to, the essential oil of *Cinnamomum zeylanicum*, which showed over 78.0% anticholinesterase and radical-scavenging activities [53]. Additionally, the essential oil of *Rosmarinus officinalis* showed antioxidant activity using the DPPH and FRAP assays [54].

So, there is an increasing demand for the development of natural antioxidants. Several assays were conducted in the current study to examine the in vitro antioxidant potentials of the *P. guajava* leaves *n*-hexane extract and the essential oil.

The antioxidant potential of the *n*-hexane extract and the essential oil was performed using different techniques as 2,2-diphenyl-1-picryl-hydrazyl-hydrate (DPPH), 2,2-azino bis (3-ethylbenzothiazoline-6-sulphonic acid) (ABTS), cupric reducing antioxidant capacity (CUPRAC), ferric reducing power (FRAP), metal chelating ability (MCA), and phosphomolybdenum (PM) assays. The findings represented in Table 2 show that the *n*-hexane extract and the essential oil have antioxidant properties in the different assays. Concerning the CUPRAC, FRAP, and MCA assays, the *n*-hexane extract revealed a higher antioxidant potential, equivalent to 70.80 ± 1.46 mg TE/g, 26.01 ± 0.97 mg TE/g, and 24.83 ± 0.35 mg EDTAE/g, respectively. By contrast, the essential oil showed more antioxidant potential in the PM assay equivalent to 2.58 ± 0.14 mmol TE/g, while *n*-hexane extract showed 2.0 ± 0.07 mmol TE/g. Regarding the DPPH and ABTS assays, none of the *n*-hexane extract or the essential oil showed any antioxidant activity. Accordingly, it can be concluded that the *n*-hexane extract and the essential oil from *P. guajava* leaves have promising antioxidant properties. The higher antioxidant potential of the *n*-hexane extract could be attributed to the presence of squalene as a major compound (9.76%), which is a well-known triterpenoid hydrocarbon with an antioxidant potential through oxygen scavenging [55]. Furthermore, *α*-Tocopherol, which is the significant antioxidant isomer of vitamin E [56] through the scavenging of free radicals, cell membrane maintenance, and structural restoration [57], is present as a major constituent in the *n*-hexane extract (8.53%). Moreover, the presence of phytosterol as *γ*-sitosterol plays an important role in the antioxidant activity of *P. guajava* [25,58]. The results were in accordance with the previous studies, where the monoterpene hydrocarbon D-limonene was reported to be a major compound in celery seed oil, which showed a high antioxidant activity using the DPPH assay [59]. Additionally, the antioxidant potential of the essential oil from *Wedelia prostrata* was attributed to the presence of a high percentage of D-limonene [60]. Regarding squalene, Kraujalis et al. (2013) reported its promising antioxidant activity in the lipophilic fraction of *Amaranthus* spp. prepared using a supercritical carbon dioxide extraction technique [61]. Moreover, it was reported that D-limonene has the ability to prevent lipidemic-oxidative stress [62,63]. Furthermore, *β*-caryophyllene was previously reported as a free-radical-scavenging agent [64,65].

The previous reports found that the antioxidant properties of the *n*-hexane extract and the essential oil of *P. guajava* was carried out using different assays, such as DPPH, ABTS, and FRAP assays and our results represented a comprehensive antioxidant profiling of the *n*-hexane extract and the essential oil of *P. guajava* available to date, using a standard equivalent way. Ashraf et al. (2016) reported the antioxidant properties of the hexane extract of *P. guajava* using the DPPH assay and found a low scavenging of free radicals (IC_50_ value = 426.8 ± 0.19 µg/mL) [25]. In another study, the antioxidant properties of the essential oil of *P. guajava* were investigated using the DPPH, ABTS, and *β*-carotene bleaching assays, which showed IC_50_ values of 17.66 ± 0.07, 19.28 ± 0.03, and 3.17 ± 0.01 μg/mL, respectively [30]. The variability in the results could be attributed to the differences in the harvest times, the maturity stage, and variations in the extraction procedure and the extracting solvent. So, these reports confirmed the antioxidant activity of *n*-hexane extract and the essential oil of *P. guajava*.

### 2.4. Enzyme Inhibitory Activity of the n-Hexane Extract and the Essential Oil Isolated from P. guajava Leaves

The enzyme inhibitory activities of the *n*-hexane extract and the essential oil were evaluated against different important enzymes, including acetylcholinesterase (AChE), butyrylcholinesterase (BChE), tyrosinase, *α*-amylase, and *α*-glucosidase. The results are represented in Table 3. It revealed that the essential oil showed a potent BChE inhibitory ability 6.85 ± 0.03 mg GALAE/g by contrast the *n*-hexane extract did not display any AChE or BChE inhibitory abilities. The strongest tyrosinase inhibition ability was determined to be the essential oil (61.70 ± 3.21 mg KAE/g) whereas the *n*-hexane extract was 33.91 ± 2.25 mg KAE/g. Regarding the anti-diabetic enzyme inhibition, the *n*-hexane extract and essential oil both displayed *α*-amylase inhibition equivalent to 0.52 ± 0.01 and 0.13 ± 0.01 mmol ACAE/g, respectively. In contrast to the *α*-amylase inhibition, the essential oil displayed a higher *α*-glucosidase inhibition equivalent to 1.49 ± 0.01 mmol ACAE/g and the *n*-hexane extract displayed less *α*-glucosidase inhibition (0.67 ± 0.03 mmol ACAE/g).

The significant BChE inhibition by the essential oil could be attributed to the presence of monoterpenes as the major components relative to the previous studies that correlate the presence of several monoterpenes with the anticholinesterase properties [65,66,67,68]. To the best of our knowledge no previous comprehensive studies were available concerning the comparative study on the enzyme inhibition of the Guava essential oil and *n*-hexane extract. Zhang et al. (2022) reported the significant *α*-amylase and *α*-glucosidase inhibitory activities of the essential oil of Guava collected from China with IC_50_ values of 13.99 ± 0.34 and 5.50 ±1.02 μg/mL, respectively [30]. Bouchoukh et al. (2019) reported the anticholinesterase properties of different extracts of Guava; the chloroform, ethyl-acetate and the *n*-butanol extracts showed AChE inhibitory activities with IC_50_ values of 177.11 ± 2.30, 56.11 ± 4.04, and 24.44 ±3.45 μg/mL, respectively; their BChE inhibitory activities were found to have IC_50_ values of >200, 44.95 ± 2.67 and 21.87 ±10.48 μg/mL, respectively [40].

Bonesi et al. (2010) reported that *trans*-caryophyllene identified in oil, showed significant BChE inhibitory activity with an IC_50_ value of 78.6 ± 1.3 μg/mL [6]. Furthermore, it acts as an antagonist to homomeric nicotinic acetylcholine receptors (α7-nAChRs) [69]. Additionally, the AChE inhibitory activity of *Artemisia annua* oil was attributed to the presence of limonene, *β*-caryophyllene, and *β*-caryophyllene oxide as the major components [70]. Zarrad et al. (2015) reported that the AChE inhibitory activity of limonene correlated with its bicyclic monoterpene hydrocarbon containing an allylic methyl group, which has an important role in its insecticidal activity [67]. A previous study by Chear et al. (2016), reported that the combination of sterols and tocopherol played an important role in the cholinesterase inhibitory activity in either AChE or BChE [71]. Recently, it was shown that *α*-tocopherol has an inhibiting effect on *α*-glucosidase being beneficial to reduce the risk factors associated with diabetes [72]. In addition, the presence of *α*-tocopherol in *Cosmos caudatus* extract revealed the potential α-glucosidase inhibitory activity [73]. You et al. (2011) reported the tyrosinase inhibitory activities of the different parts and different extracts of Guava. The leaves, acetone, ethanol, methanol, and water extracts showed tyrosinase inhibition by 49.67 ± 0.58, 69.56 ± 1.38, 47.33 ± 1.84, and 44.78 ± 1.75%, respectively [74]. Development of tyrosinase inhibitors from natural sources is in great demand due to their low side effects and higher efficacy making natural tyrosinase inhibitors a good candidate for the incorporation in hypopigmenting agents. It is worth mentioning that tyrosinase plays an important role in melanin synthesis [75,76,77].

### 2.5. Molecular Docking

**Figure 4:**
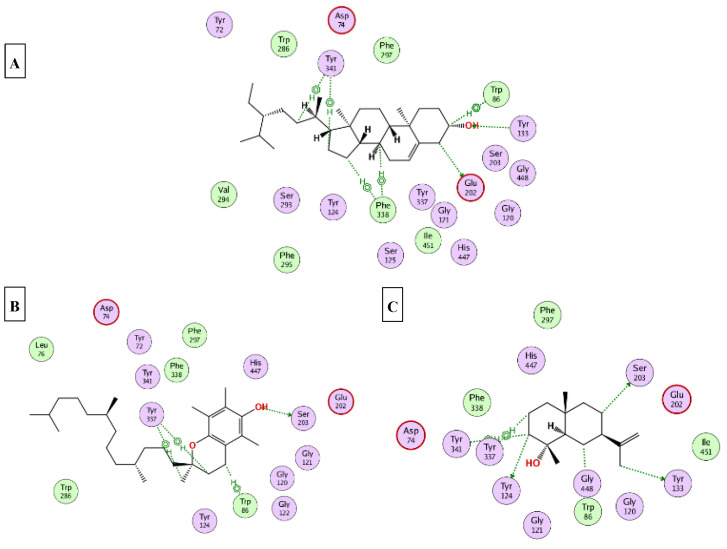
2D binding modes of *γ*-sitosterol (**A**); α-tocopherol (**B**); selin-11-en-4-*α*-ol (**C**) to the active binding sites of AChE.

**Figure 5:**
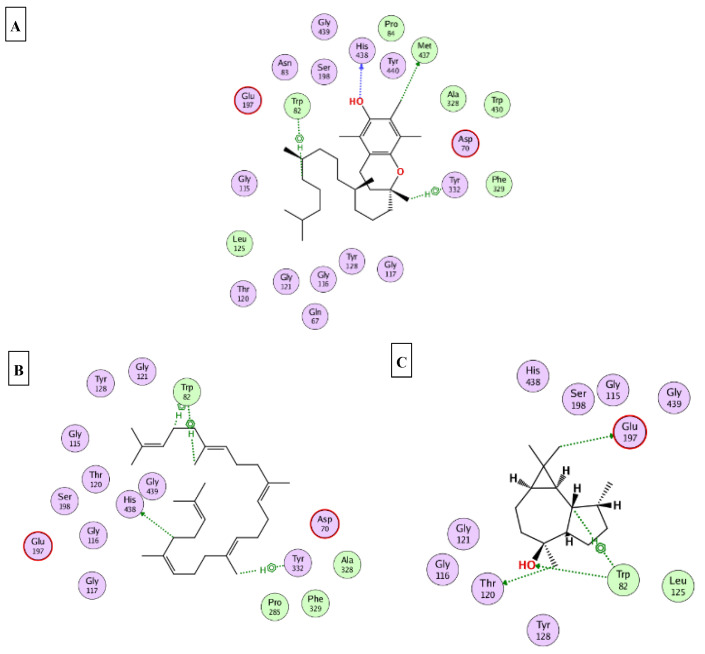
2D binding modes of *α*-tocopherol (**A**); squalene (**B**); viridiflorol (**C**) to the active binding sites of BChE.

**Figure 6:**
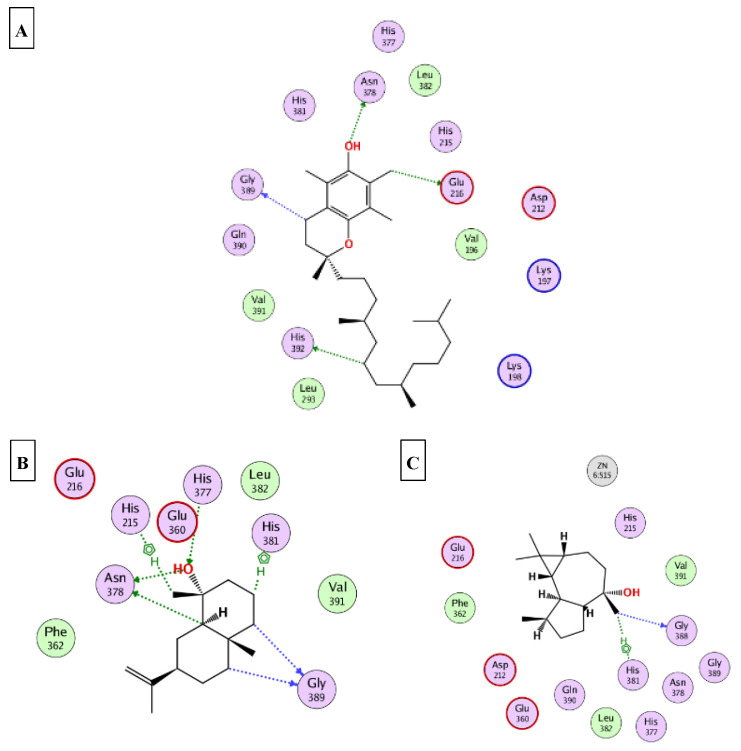
2D binding modes of *α*-tocopherol (**A**); selin-11-en-4-*α*-ol (**B**); viridiflorol (**C**) to the active binding sites of tyrosinase.

**Figure 7:**
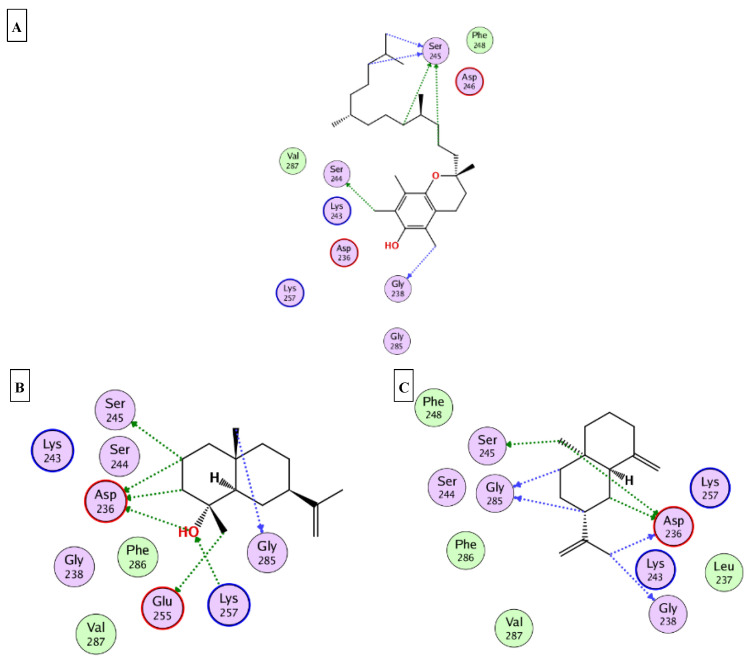
2D binding modes of *α*-tocopherol (**A**); selin-11-en-4-*α*-ol (**B**); *β*-selinene (**C**) to the active binding sites of *α*-amylase.

**Figure 8:**
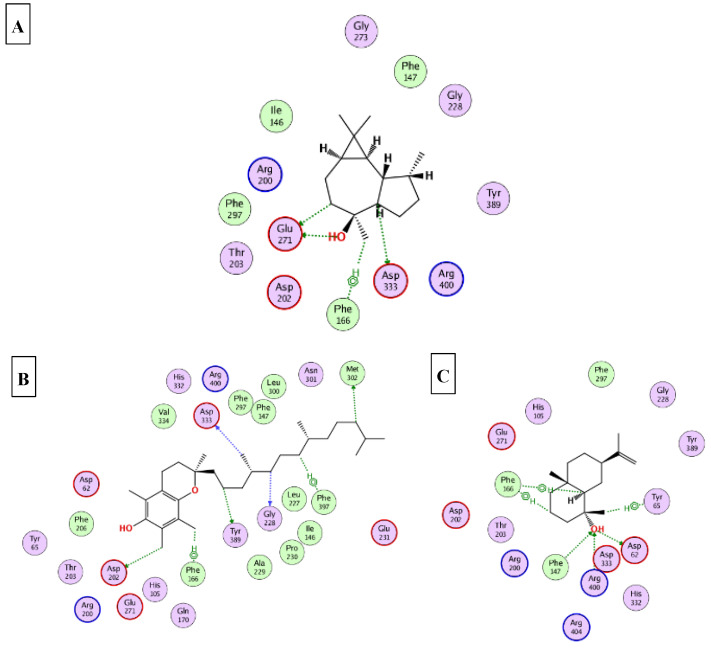
2D binding modes of viridiflorol (**A**); *α*-tocopherol (**B**); selin-11-en-4-*α*-ol (**C**) to the active binding sites of *α*-glucosidase.

This part was conducted to investigate the possible mechanism of action in which the ten major compounds (D-limonene, *β*-caryophyllene, *β*-selinene, viridiflorol, 1-epi-cubenol, caryophylla-4(12),8(13)-dien-5*α*-ol, selin-11-en-4-*α*-ol, squalene, *α*-tocopherol, and *γ*-sitosterol) exert their biological effects. Accordingly, the 3D structures of AChE, BchE, tyrosinase, *α*-amylase, and *α*-glucosidase were downloaded from the protein data bank using the following IDs: 7D9O, 6ESJ, 5M8Q, 4GQQ, and 3WY2, respectively. After that, the ten major compounds were docked into the active site vicinity of the five enzymes. Interestingly, all the compounds achieved acceptable binding scores upon docking with the five targets (Table 4). In the docking of AChE, *γ*-sitosterol, *α*-tocopherol, and selin-11-en-4-α-ol the best scores for docking were achieved −15.4, −14.2, and −13.4 Kcal/Mol, respectively. As Figure 4 reveals, *γ*-sitosterol interacted with AChE through mixed hydrophobic and hydrogen bond interactions with Tyr341, Phe338, Trp86, Tyr133, and Glu203. Moreover, *α*-tocopherol interacted with Trp86, Ser203 and 337; selin-11-en-4-α-ol interacted with Tyr124, Tyr133, Ser203, Tyr337, and Tyr341. In the docking of BChE, *α*-tocopherol, squalene, and viridiflorol achieved the best docking scores −13.9, −11.1, and −10.7 Kcal/Mol, respectively. As depicted Figure 5 *α*-tocopherol bound to BChE through interactions with Trp82, Tyr332, Met437, and His438; squalene interacted with Trp82, Tyr332, and His438; viridiflorol interacted with Trp82, Thr120, and Glu197. In the docking of tyrosinase, *α*-tocopherol, selin-11-en-4-α-ol, and viridiflorol achieved the best docking scores −9.5, −9.5, and −9.3 Kcal/Mol, respectively. Figure 6 reveals the interaction of the best three compounds with tyrosinase in which, *α*-tocopherol interacted with Glu216, Asn378, Gly389, and His392, selin-11-en-4-α-ol interacted with His215, His377, Asn378, His381, and Gly389 and viridiflorol interacted with His318 and Gly388. Regarding the docking of *α*-amylase, *α*-tocopherol, selin-11-en-4-α-ol, and *β*-selinene achieved the best docking scores −8.9, −7.8, and −7.7 Kcal/Mol, respectively. Inspecting Figure 7, *α*-tocopherol was able to interact with the residues of *α*-amylase through binding with Gly238, Ser244, and Ser245; selin-11-en-4-α-ol interacted with Asp236, Ser245, Glu255, Lys257, and Gly285; *β*-selinene interacted with Asp236, Ser245, Gly238, and Gly285. In the docking of *α*-glucosidase, viridiflorol, *α*-tocopherol, and selin-11-en-4-α-ol achieved the best docking scores −13.6, −12.5, and −12.5 Kcal/Mol, respectively. Figure 8 shows the interaction of the best three compounds with *α*-glucosidase in which, viridiflorol interacted with Phe166, Glu271, and Asp333; *α*-tocopherol interacted with Phe166, Asp202, Gly228, Met302, Tyr389, Phe397, and Asp333; selin-11-en-4-α-ol interacted with Phe166, Asp62, Tyr65, Phe147, and Arg400. In conclusion, the docking results supported and justified the biological results giving rise to a synergetic effect for all the components of the *n*-hexane extract and the essential oil.

## 3. Materials and Methods

### 3.1. Plant Material

Fresh leaves of *P. guajava* Linn. were collected from the Medicinal Plant Research Station, Pharmacognosy Department, Faculty of Pharmacy, Ain Shams University, Cairo, Egypt, in October 2021. The plant was authenticated by Professor Usama K. Abdel Hameed, Department of Botany, Faculty of Science, Ain Shams University, Cairo, Egypt. A voucher specimen, PHG-P-PG-409, was deposited at the Pharmacognosy Department, Faculty of Pharmacy, Ain Shams University, Cairo, Egypt.

### 3.2. Isolation of the Essential Oil

The fresh leaves were finely cut and hydrodistilled for 5 h using a Clevenger apparatus. The oil obtained is colorless with a pleasant aroma; the average yield was 0.2% (*v*/*w*). It was isolated and kept in a sealed dark glass vial at −4 °C until the GC/MS analysis was performed. 

### 3.3. Preparation of the n-Hexane Extract

The dried leaves of *Psidium guajava* Linn. (100 g) were extracted with *n*-hexane three times separately. The filtrate was completely evaporated in vacuo at 40 °C until dryness to obtain the dried residue of the *n*-hexane extract (3.2 g). The extract was stored in a tight container and stored in a refrigerator for further analysis.

### 3.4. Gas Chromatography/Mass Spectrometry (GC/MS) Analysis

Gas chromatography/Mass spectrometry (GC/MS) analysis was carried out on a Shimadzu GCMS-QP 2010 chromatograph (Kyoto, Japan) with Rtx-1MS capillary column (30 m × 0.25 mm i.d. × 0.25 μm film thickness; Restek, Bellefonte, PA, USA). The oven temperature was kept at 45 °C for 2 min (isothermal), programmed to 30 °C at a rate of 5 °C/min, and kept constant at 300 °C for 5 min (isothermal); injector temperature was 250 °C. The carrier gas used was helium, with a flow rate set at 1.40 mL/min. The diluted samples (1% *v*/*v*) were injected with a split ratio of 15:1 and the injected volume was 1 μL. The MS operating parameters were as follows: interface temperature 280 °C, ion-source temperature 220 °C, EI mode 70 eV, scan range 35–500 amu. Identification of the volatile constituents was made based on their retention indices, matching their fragmentation patterns with the NIST Mass Spectral Library, the Wiley library database, and the published data in the literature [78,79,80,81]. Retention indices (RI) were calculated relative to the homologous series of *n*-alkanes (C8–C30) and injected under the same conditions.

### 3.5. Compounds Identification

The identification was accomplished by comparing the Kovats retention index and the mass spectrometric data (molecular ion peaks and fragmentation patterns), to those recorded in the NIST Mass Spectral Library and other published data for the reference compounds under similar conditions [56,78,79,80,81,82,83].

### 3.6. Total Phenolic and Flavonoid Content

The total phenolic and flavonoid contents were determined using the Folin–Ciocalteu and AlCl_3_ tests, respectively [45]. Results were presented as gallic acid equivalents (mg GAEs/g dry extract) and rutin equivalents (mg REs/g dry extract) for the assays. All experimental details are given in Supplementary Materials.

### 3.7. Antioxidant and Enzyme Inhibitory Assays

The antioxidant assays were performed using methods that have been previously reported [84,85]. Trolox and EDTA were used as positive controls in the antioxidant assays. The antioxidant potential was calculated as follows: mg Trolox equivalents (TE)/g extract in the 2,2-diphenyl-1-picrylhydrazyl (DPPH) and 2,2’-azino-bis(3-ethylbenzothiazoline-6-sulfonic acid) (ABTS) radical scavenging tests; cupric reducing antioxidant capacity (CUPRAC) and ferric reducing antioxidant power (FRAP), mmol TE/g extract in phosphomolybdenum assay, and mg ethylenediaminetetraacetic acid equivalents (EDTAE)/g extract in metal chelating assay (MCA). All experimental details for the antioxidant assays are given in Supplementary Materials.

The enzyme inhibition experiments were performed based on previously described procedures [84,85]. Standard inhibitors were used as positive controls (galanthamine for cholinesterases; kojic acid for tyrosinase; acarbose for amylase and glucosidase) in the enzyme inhibitory assays. Amylase and glucosidase inhibition were expressed as mmol acarbose equivalents (ACAE)/g extract, while acetylcholinesterase (AChE) and butyrylcholinesterase (BChE) inhibition was expressed as mg galanthamine equivalents (GALAE)/g extract. Tyrosinase inhibition was expressed as mg kojic acid equivalents (KAE)/g extract. All experimental details for the enzyme inhibitory assays are given in Supplementary Materials.

### 3.8. Molecular Docking

The X-ray 3D structures of AChE, BChE, tyrosinase, *α*-amylase, and *α*-glucosidase were downloaded from the protein data bank “www.pdb.org (accessed on 3 August 2022)” using the following IDs: 7D9O, 6ESJ, 5M8Q, 4GQQ, and 3WY2 [86,87,88,89,90], respectively. All the docking studies were conducted using MOE 2019 [91], which was also used to generate the 2D interaction diagrams between the docked ligands and their potential targets. The ten identified major compounds were prepared using the default parameters and saved in a single MDB file. The active site of each target was determined from the binding of the corresponding co-crystalized ligand. Finally, the docking was finalized through docking the MDB file containing the ten major compounds into the active site of the five enzymes.

## 4. Conclusions

Chemical investigation of *Pisidium guava* have proven that this plant species contains a variety of volatile components present in the *n*-hexane extract and the essential oil, as well as their antioxidant and enzyme inhibitory activities supported with an in-silico study. The GC/MS analysis revealed that the *n*-hexane extract is a rich source of squalene (9.76%), *α*-tocopherol (8.53%), and *γ*-sitosterol (3.90%) that correlates with its antioxidant potential in the CUPRAC, FRAP, and MCA assays. On the other hand, the essential oil was enriched with monoterpenes and sesquiterpenes, especially D-limonene (36.68%) and viridiflorol (9.68%), which correlates with its antioxidant potential in different assays along with its potency as a BChE and a tyrosinase inhibitor. Furthermore, the *n*-hexane extract and the essential oil showed relevant *α*-amylase and *α*-glucosidase inhibitory activities. Furthermore, the major compounds achieved promising docking scores in the active sites of the tested target enzymes. According to these findings and the previous studies, the *n*-hexane extract and essential oil of *P. guajava* can be considered a promising candidate for the development of novel therapeutic agents for the management of Alzheimer’s, diabetes mellitus, and oxidative stress disorders. Moreover, their efficacy as tyrosinase inhibitors allows them to be incorporated in the development of hypopigmenting agents. However, further investigations should be conducted concerning the pharmacodynamics as well as the pharmacokinetics pathways accompanied with the in vivo studies.

## Figures and Tables

**Table 1:** Chemical composition (%) of the *n*-hexane extract (PGH) and essential oil (PGO) isolated from *Psidium guajava* leaves using GC/MS analysis.

No.	Rt _(min)_	Compound	RI_Exp_. ^a^	RI_Lit_ ^b^	Molecular Formula	Content (%)	
						PGH	PGO
1	7.16	*α*-Pinene	931	931	C_10_H_16_	-	0.99
2	8.99	*n*-Decane	999	1000	C_10_H_22_	1.44	-
3	9.94	*p*-Cymene	1024	1024	C_10_H_14_	-	0.13
4	10.09	D-Limonene	1029	1029	C_10_H_16_	4.83	36.68
5	12.15	*n*-Undecane	1099	1100	C_11_H_24_	3.61	-
6	14.15	2-Methylundecane	1164	1165	C_12_H_26_	0.70	-
7	14.98	*trans*-*p*-Mentha-1(7),8-dien-2-ol	1188	1185	C_10_H_16_O	-	0.50
8	15.07	*α*-Terpineol	1191	1189	C_10_H_18_O	-	0.64
9	15.25	*n*-Dodecane	1199	1200	C_12_H_26_	4.15	-
10	15.67	3,6-Dimethylundecane	1213	1210	C_13_H_28_	0.58	-
11	15.95	*trans*-Carveol	1221	1220	C_10_H_16_O	-	0.15
12	16.20	*cis*-*p*-Mentha-1(7),8-dien-2-ol	1229	1235	C_10_H_16_O	-	0.52
13	18.21	*n*-Tridecane	1299	1300	C_13_H_28_	0.45	-
14	20.43	*α*-Copaene	1378	1376	C_15_H_24_	0.51	0.59
15	21.65	*β*-Caryophyllene	1424	1424	C_15_H_24_	3.80	8.41
16	22.16	Alloaromadendrene	1443	1442	C_15_H_24_	0.42	1.60
17	22.54	Humulene (*α*-Caryophyllene)	1458	1455	C_15_H_24_	-	1.00
18	22.74	*epi*-*β*-Caryophyllene	1465	1466	C_15_H_24_	-	0.37
19	23.13	*γ*-Muurolene	1480	1479	C_15_H_24_	-	0.40
20	23.41	*β*-Selinene	1491	1486	C_15_H_24_	1.22	4.10
21	23.64	*β*-Guaiene	1500	1500	C_15_H_24_	1.05	2.94
22	23.75	*α*-Bisabolene	1504	1506	C_15_H_24_	-	1.12
23	23.92	*β* -Bisabolene	1511	1512	C_15_H_24_	-	1.33
24	24.11	*γ*-Cadinene	1519	1513	C_15_H_24_	-	0.23
25	24.33	*cis*-Calamenene	1528	1529	C_15_H_22_	-	0.67
26	24.56	Cubenene	1537	1533	C_15_H_24_	1.75	0.34
27	24.79	Ledol	1547	1549	C_15_H_26_O	1.27	-
28	25.28	Dodecanoic acid	1566	1566	C_12_H_24_O	1.20	0.88
29	25.54	Caryophyllene alcohol	1577	1572	C_15_H_26_O	-	0.35
30	25.68	Caryophyllene oxide	1582	1583	C_15_H_24_O	-	0.20
31	25.89	Viridiflorol	1591	1592	C_15_H_26_O	0.95	9.68
32	26.09	Globulol	1598	1590	C_15_H_26_O	-	0.49
33	26.15	Benzene, (1-methylnonyl)-	1602	1616	C_16_H_26_	1.26	-
34	26.42	*β*-Atlantol	1613	1608	C_15_H_24_O	-	1.29
35	26.51	*β*-Himachalene oxide	1616	1616	C_15_H_24_O	-	0.81
36	26.60	Humulene epoxide II	1620	1620	C_15_H_24_O	-	0.61
37	26.70	Alloaromadendrene oxide-(1)	1624	1625	C_15_H_24_O	-	0.26
38	26.82	*γ*-Eudesmol	1630	1632	C_15_H_26_O	2.08	0.39
39	26.94	1-*epi*-Cubenol	1635	1630	C_15_H_26_O	4.51	1.38
40	27.08	Caryophylla-4(12),8(13)-dien-5*ꞵ*-ol	1640	1640	C_15_H_24_O	-	1.87
41	27.17	Caryophylla-4(12),8(13)-dien-5*α*-ol	1645	1641	C_15_H_24_O	3.64	6.48
42	27.37	*α*-Cadinol	1653	1654	C_15_H_26_O	-	0.97
43	27.61	Selin-11-en-4-*α*-ol	1663	1659	C_15_H_26_O	-	6.35
44	27.69	Benzene, (1-ethylnonyl)-	1668	1670	C_17_H_28_	2.93	-
45	27.83	*epi*-*β*-Bisabolol	1672	1672	C_15_H_26_O	-	0.69
46	27.96	Khusilol	1678	1676	C_14_H_20_O	-	1.99
47	28.19	*α*-Bisabolone oxide A	1688	1686	C_14_H_22_O_2_	-	0.55
48	28.29	11*α*H-Himachal-4-en-1*β*-ol	1692	1699	C_15_H_26_O	-	1.35
49	28.55	Benzene, (1-methyldecyl)-	1704	1715	C_17_H_28_	3.46	-
50	29.14	Benzene, (1-pentylheptyl)-	1728	1718	C_18_H_30_	3.60	-
51	29.25	Benzene, (1-butyloctyl)-	1733	1725	C_18_H_30_	3.71	-
52	29.52	Benzene, (1-propylnonyl)-	1744	1741	C_18_H_30_	2.70	-
53	30.01	Benzene, (1-ethyldecyl)-	1764	1767	C_18_H_30_	2.55	-
54	30.85	Benzene, (1-methylundecyl)-	1799	1797	C_18_H_30_	2.94	-
55	31.31	Benzene, (1-pentyloctyl)-	1822	1819	C_19_H_32_	3.19	-
56	31.46	Benzene, (1-butylnonyl)-	1830	1825	C_19_H_32_	2.65	-
57	31.73	Benzene, (1-propyldecyl)-	1844	1838	C_19_H_32_	1.76	-
58	32.23	Benzene, (1-ethylundecyl)-	1870	1866	C_19_H_32_	1.50	-
59	33.05	Benzene, (1-methyldodecyl)-	1912	1911	C_19_H_32_	1.76	-
60	37.04	Phytol	2115	2114	C_20_H_40_O	1.90	-
61	38.37	Palmitic acid, butyl ester	2187	2188	C_20_H_40_O_2_	0.75	-
62	41.38	Eicosanoic acid, methyl ester	2358	2339	C_21_H_42_O_2_	0.64	-
63	41.52	Linolenic acid, ethyl ester	2366	-	C_20_H_34_O_2_	0.83	-
64	48.77	Squalene	2834	2835	C_30_H_50_	9.76	-
65	50.27	Hexacosanoic acid, methyl ester	2942	2940	C_27_H_54_O_2_	0.41	-
66	53.07	*α*-Tocopherol	3152	3149	C_29_H_50_O_2_	8.53	-
67	55.98	*γ*-Sitosterol	3352	3351	C_29_H_50_O	3.90	-
Monoterpene hydrocarbons						4.83	37.80
Oxygenated monoterpenes						-	1.81
Sesquiterpene hydrocarbons						8.75	23.10
Oxygenated sesquiterpenes						13.65	36.59
Diterpenoids						1.90	-
Triterpenoids						9.76	-
Sterols						3.90	-
Aromatic Hydrocarbons						34.01	-
Aliphatic Hydrocarbons						10.93	-
Fatty acids and fatty acids derivatives						2.63	-
Others						8.53	-
Total identified compounds						98.89	99.30

Compounds listed in order of their elution on DB-5 GC column. Identification was based on comparison of the compounds mass spectral data (MS) and retention indices (RI) with those of NIST Mass Spectral Library (2011), Wiley Registry of Mass Spectral Data 8th edition and the literature [28,30]. ^a^ Retention index calculated experimentally on Rtx-1MS column relative to *n*-alkane series (C8–C28). ^b^ Published retention indices.

**Table 2:** Antioxidant potential of the *n*-hexane extract and essential oil isolated from *P. guajava* leaves.

Samples	DPPH	ABTS	CUPRAC	FRAP	MCA	PM
	(mg TE/g)	(mg TE/g)	(mg TE/g)	(mg TE/g)	(mg EDTAE/g)	(mmol TE/g)
*n*-Hexane extract	n.a.	n.a.	70.80 ± 1.46	26.01 ± 0.97	24.83 ± 0.35	2.0 ± 0.07
Essential oil	n.a.	n.a.	18.17 ± 0.08	12.08 ± 0.17	9.02 ± 1.2	2.58 ± 0.14

Values expressed as means ± S.D. of three parallel measurements. Trolox equivalent (TE); Ethylenediaminetetraacetic acid equivalent (EDTAE); not active (n.a.).

**Table 3:** Enzyme inhibitory effects of the *n*-hexane extract and the essential oil isolated from *P. guajava* leaves.

Samples	AChE Inhibition	BChE Inhibition	Tyrosinase Inhibition	*α*-Amylase Inhibition	*α*-Glucosidase Inhibition
	(mg GALAE/g)	(mg GALAE/g)	(mg KAE/g)	(mmol ACAE/g)	(mmol ACAE/g)
*n*-Hexane extract	n.a.	n.a.	33.91 ± 2.25	0.52 ± 0.01	0.67 ± 0.03
Essential oil	n.a.	6.85 ± 0.03	61.70 ± 3.21	0.13 ± 0.01	1.49 ± 0.01

Values expressed as means ± S.D. of three parallel measurements. Galanthamine equivalent (GALAE); Kojic acid equivalent (KAE); Acarbose equivalent (ACAE); not active (n.a.).

**Table 4:** The docking scores achieved by the major identified compounds against different enzymes.

Compound		Docking Scores Kcal/mol			
	AChE 7D9O	BChE 6ESJ	Tyrosinase 5M8Q	*α*-Amylase 4GQQ	*α*-Glucosidase 3WY2
D-Limonene	−9.2	−7.2	−7.3	−6.1	−7.9
*β*-Caryophyllene	−9.3	−8.7	−7.2	−6.3	−7.9
*β*-Selinene	−9.5	−7.7	−6.8	−7.7	−7.6
Viridiflorol	−11.4	−10.7	−9.3	−6.3	−13.6
1-*epi*-Cubenol	−10.2	−8.8	−6.9	−6.4	−8.3
Caryophylla-4(12),8(13)-dien-5*α*-ol	−11.4	−9.1	−7.6	−6.8	−8.9
Selin-11-en-4-*α*-ol	−13.4	−9.3	−9.5	−7.8	−12.5
Squalene	−12.3	−11.1	−7.9	−6.4	−9.1
*α*-Tocopherol	−14.2	−13.9	−9.5	−8.9	−12.5
*γ*-Sitosterol	−15.4	−9.4	−8.2	−7.1	−9.3

## Data Availability

Data are available upon request.

## Supplementary Materials

The following supporting information can be downloaded at: https://www.mdpi.com/article/10.3390/molecules27248979/s1.
